# A unifying account of loss aversion and loneliness: from neurocognitive bases to affective dysfunction

**DOI:** 10.3389/fpsyg.2026.1777535

**Published:** 2026-02-09

**Authors:** Maria Arioli, Zaira Cattaneo, Nicola Canessa

**Affiliations:** 1 Department of Human and Social Sciences, University of Bergamo, Bergamo, Italy; 2 IUSS Cognitive Neuroscience (ICoN) Center, Scuola Universitaria Superiore IUSS, Pavia, Italy; 3 Cognitive Neuroscience Laboratory of Pavia Institute, Istituti Clinici Scientifici Maugeri IRCCS, Pavia, Italy

**Keywords:** intervention, loneliness, loss aversion, negativity bias, self-preservation, treatment

## Abstract

In this perspective paper we advance the hypothesis that loss aversion (the general tendency to weigh losses significantly more than equivalent gains) and loneliness (the distress arising from perceived social deficiency and isolation) share a fundamental basis in negativity bias and partially overlapping neuro-cognitive mechanisms. Although traditionally studied separately, we argue that both phenomena reflect heightened sensitivity to negative information, expressed in distinctive attentional and expectancy biases, and in opposite response patterns in striatal and insular neural systems processing rewards and negative affects, respectively. Moreover, both phenomena are associated with individual differences in emotion regulation and cognitive control, reflected in altered amygdala-prefrontal cortex connectivity. We propose that - when exacerbated - these shared behavioral and neural patterns may contribute to the affective dysfunction observed in depression, thereby helping explain the robust association of this condition with both loss aversion and loneliness. By integrating evidence from experimental psychology, cognitive neuroscience and clinical research, we outline the shared underpinnings of loss aversion and loneliness while also delineating their theoretical and experimental differences. This unified framework offers novel insights into the cognitive and neural mechanisms supporting self-preservation, and motivates future interdisciplinary investigation linking decision-making with social attitudes and interactions.

## 1 Introduction: *bad is stronger than good*

In a dynamic, unpredictable, stimulus-rich environment that includes harmful and dangerous elements, the development and learning of defense strategies are crucial for increasing an individual’s probability of survival and fitness. The individual’s aim may therefore become self-preservation, to limit potential damage through strategies that allow for better environmental understanding and, accordingly, the prediction and prevention of possible dangers (*exploration*), but also through basic, habitual survival strategies (*exploitation*) (Mobbs et al., 2024). Consistent with this goal, potentially negative and harmful events are known to capture attention more readily and to be weighted more heavily than positive events during evaluative, decisional, and subsequent action-implementation processes (Norris, 2021; see also Lazarus, 2021 for a different evolutionary perspective). This asymmetry—often summarized by the general principle “bad is stronger than good”—is commonly referred to as the *negativity bias* (Baumeister et al., 2001).

Despite considerable individual differences in its behavioral expression (Norris et al., 2011), most individuals display this bias across multiple contexts, in response to broad categories of stimuli (e.g., images, sounds, words; Norris, 2021), and over the entire circle of lifespan from infancy (Chae and Song, 2018; Xie et al., 2019) to older adulthood (Norris, 2021), although some evidence suggests the emergence of a positivity effect in late adulthood (Carstensen and DeLiema, 2018). Neuroscientific evidence points to a neural basis for the negativity bias, reflected in heightened responses to negative compared with positive stimuli (Huang and Luo, 2006; Yoshida et al., 2021).

In particular, manifestations of the negativity bias can be observed in two prominent phenomena in cognitive neuroscience such as loneliness (Bellucci et al., 2025) and loss aversion (Norris, 2021). Although typically conceptualized and investigated as distinct constructs within separate research traditions, in this perspective paper we advance a novel conceptual link between them, arguing that they may involve partially shared neuro-cognitive mechanisms.

## 2 Loss aversion: *losses loom larger than gains*

**Figure 1:**
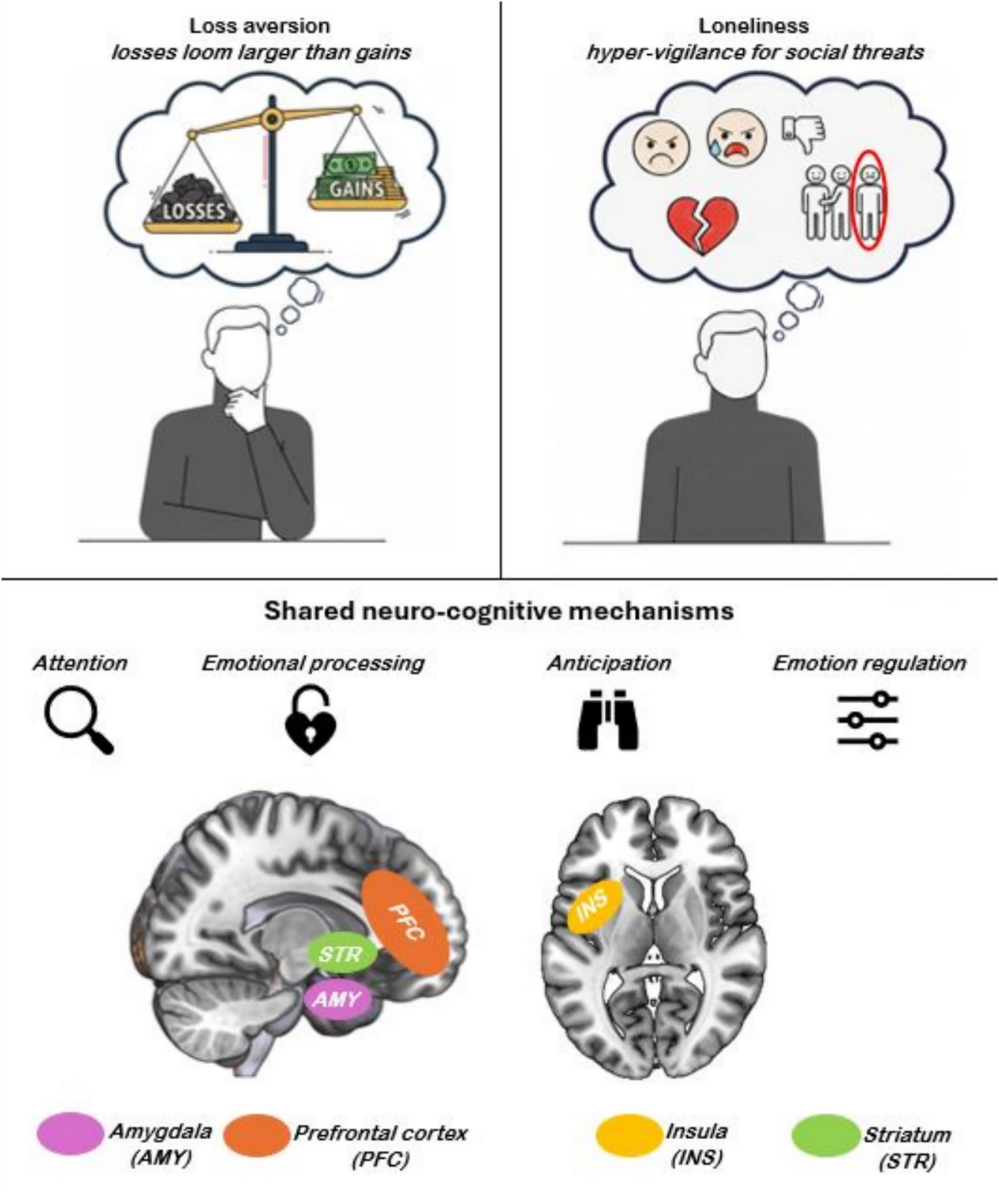
Graphical representation of the negativity bias in loss aversion (top left) and loneliness (top right), along with the shared neuro-cognitive mechanisms possibly underlying both phenomena (bottom).

Loss aversion is a well-established construct in behavioral economics and psychology referring to the greater psychological impact of prospective losses relative to equivalent gains in decision-making under risk (Kahneman and Tversky, 2013; Kahneman et al., 1991; see Figure 1 top left). Originally described in the context of economic decision-making (Kahneman and Tversky, 2013), it is typically investigated through controlled experimental paradigms involving mixed-gambles (e.g., Arioli et al., 2023) in which participants decide whether to accept or reject gambles offering an equal 50% probability of either winning or losing varying monetary amounts. Identifying the indifference point in this mixed-gamble task allows to estimate a “loss aversion” coefficient, commonly denoted as lambda (*λ*; Molins and Serrano, 2019; Sokol-Hessner and Rutledge, 2019), that captures the extent to which losses are overweighted relative to gains during valuation. Robust empirical evidence estimates λ at around 2 (1.8–2.1 in Brown et al.’s, 2024 meta-analysis; but see also Yechiam and Zeif, 2025 and Walasek et al., 2024), suggesting that losses exert roughly twice the subjective impact of equivalent gains. From an evolutionary perspective, loss aversion may have evolved as an adaptive self-protective mechanism (Li et al., 2012), because the costs of events such as injury or resource depletion, would have outweighed the benefits of comparable gains.

Extending beyond behavioral economics, loss aversion appears to shape decision-making across multiple domains, including non-monetary contexts, confirming its role in broader valuation processes (Rick, 2011). Consistent with the view that it reflects a deep-seated tendency, individual differences in loss aversion have been associated with multiple neuro-physiological markers, including metrics of arousal (Sokol-Hessner et al., 2009; Sheng et al., 2020), interoception (Sokol-Hessner et al., 2015), as well as brain activity (Canessa et al., 2013, 2017; Tom et al., 2007), connectivity (Arioli et al., 2025c) and structure (Arioli et al., 2025a).

## 3 Loneliness: *hyper-vigilance for social threats*

Loneliness refers to the distressing feeling that one’s social relationships fall short of what one desires (Perlman and Peplau, 1981; for a recent review see Bellucci et al., 2025), reflecting in the subjective sense of social isolation and disconnection (see Figure 1 top right). Loneliness is typically evaluated with variants of the UCLA (University of California, Los Angeles) Loneliness Scale (Russell et al., 1978), a self-report questionnaire including items assessing perceived deficits in close and supportive relationships, such as the perceived lack of trusted confidant(s) and of mutual aid, protection, and acceptance. Neuropsychological research has typically examined this condition and its effects by contrasting individuals with high vs. low loneliness (Cacioppo et al., 2015), and/or by testing associations between loneliness and specific behavioral or neural responses (Arioli et al., 2025b). While most individuals do not report feeling lonely at a given moment, studies on older adults in high-income countries suggest that approximately 1 in 4 experience some degree of loneliness at least occasionally (Chawla et al., 2021; see also Cacioppo and Cacioppo, 2018 for an overview). Previous research has shown that the experience of loneliness is significantly shaped both by genetic predispositions (Abdellaoui et al., 2019) and environmental influences (e.g., supportive co-housing that does not undermine independence; Bower et al., 2023).

Since social interactions can enhance survival and reproductive success but also entail risks such as exposure to violence, the motivating effect of loneliness towards social connection is expected to reflect in the implicit hyper-vigilance to negative social cues, that may then be coded as possible social threats signaling rejection or isolation (Spithoven et al., 2017). The recognition of such side-effects of social connection shaped the view that loneliness evolved not merely as a biological drive to reconnect, but as an adaptive mechanism that triggers implicit vigilance and the avoidance of social threats to prevent social damage (Cacioppo and Cacioppo, 2018). This tendency is often grounded in the aforementioned evolutionary cost–benefit logic, i.e., that the costs of failing to detect a potential threat outweigh those of missing a social opportunity (Cacioppo et al., 2014). Accordingly, loneliness may automatically trigger heightened vigilance for - and avoidance of—negative social events, alongside more self-focused and protective behavioral tendencies (Cacioppo and Cacioppo, 2018), which can paradoxically hamper social reconnection (Cacioppo and Hawkley, 2009; Holt-Lunstad et al., 2015; Bzdok and Dunbar, 2020). Converging neuroscientific evidence provides complementary support, showing that the experience of loneliness is associated with distinctive activation patterns in brain networks associated with attentional and socio-cognitive processing (Lam et al., 2021).

## 4 The unexplored link between loss aversion and loneliness: new insights into shared mechanisms

Any possible link between loss aversion and loneliness depends on the presence of shared underlying mechanisms. We therefore discuss the cognitive processes involved in both phenomena, their neural correlates (see Figure 1 bottom), and, finally, how their extreme manifestation relates to functional impairments commonly associated with depression.

### 4.1 Cognitive mechanisms

Psychological research suggests that both loss aversion and loneliness are characterized by attentional and anticipatory biases toward negative information. There is evidence that, compared to non-lonely individuals, lonelier ones show higher sensitivity to (Bangee et al., 2014; Cacioppo et al., 2016) and enhanced memory for (Teneva and Lemay, 2020) negative social stimuli. This negativity bias also involves social expectations, as shown by lonelier individuals’ tendency to form more negative impressions of others and, accordingly, to expect less trustworthy behaviors in future social exchanges (Bellucci and Park, 2023). Similarly, loss aversion appears to be associated with negatively biased attentional processing and outcome anticipation, with higher *λ* values predicting greater attention to losses than to gains (Pachur et al., 2018), as well as overestimation of both the impact of losses and one’s tendency to dwell on these losses (Kermer et al., 2006).

These patterns have also been associated with alterations in emotion regulation and cognitive control in both loneliness and loss aversion, consistent with a reduced capacity to downregulate affective responses to negative cues. The available literature shows that loneliness is associated with strong negative affect, poor emotion regulation, and inflexible emotion-focused coping, such that lonely people may struggle to alleviate their negative feelings (Cacioppo and Hawkley, 2009; Wong et al., 2022). In the case of loss aversion, intentional cognitive regulation strategies have been reported to reduce both behavioral loss aversion and the physiological arousal to losses relative to gains (Sokol-Hessner et al., 2009), supporting the view that loss aversion can be attenuated under successful emotion regulation.

### 4.2 Neural mechanisms

Since both loss aversion and loneliness are characterized by negatively biased processing, we hypothesize convergent response profiles within affective brain systems that preferentially encode negative relative to positive stimuli and/or experiences. The available literature shows that the reward/positive-valence system is grounded in the striatum (Doré et al., 2017; Haber, 2017), while its negatively-valenced counterpart includes the amygdala and insula (Carretie et al., 2009; Shiba et al., 2017). Neuroimaging studies on loss aversion have indeed shown, in both these systems, distinctive patterns of “neural loss aversion” (NLA) i.e., asymmetric bidirectional responses to losses vs. gains (Molins and Serrano, 2019). The posterior insular cortex shows greater activation during loss anticipation than deactivation during gain anticipation (loss-oriented NLA; Canessa et al., 2013; Fukunaga et al., 2012), whereas the reward system (particularly ventral striatum and midcingulate cortex) shows the opposite profile of stronger deactivation by anticipated losses than activation by anticipated gains (gain-oriented NLA; Schulreich et al., 2020; Tom et al., 2007). Notably, preliminary evidence highlights a similar bidirectional pattern for loneliness, that is negatively associated with striatal responses, and positively associated with insular activity (Luo and Shao, 2023).

Beyond altered affective processing, both loss aversion and loneliness have been associated with difficulties in emotion regulation that may sustain the negativity bias. Loneliness has been found to be negatively associated with connectivity between the amygdala and superior frontal gyrus (Wong et al., 2016), consistent with reduced prefrontal regulation of amygdala activity and, accordingly, heightened amygdala responses and negative emotion biases (Luo and Shao, 2023). High loneliness has been also associated with broader alterations in the functional connectivity of cognitive control networks (Layden et al., 2017; Shao et al., 2020; Tian et al., 2017). While some evidence points to compensatory recruitment within cognitive control networks, prolonged reliance on such mechanisms may deplete cognitive resources and ultimately contribute to emotional dysregulation (Wong et al., 2022). Similarly, loss aversion appears to be inversely related to emotion regulation skills, such that successful control over loss averse behavior is associated with reduced amygdala responses to losses alongside increased prefrontal activity (Sokol-Hessner et al., 2013).

### 4.3 Impaired mechanisms: depression

These shared behavioral and neural signatures of loneliness and loss aversion are consistent with a biased processing of negative stimuli, expectations, and memories, and have been associated with alterations in emotion regulation and cognitive control. At their highest degree, such complex and distinctive patterns may contribute to clinical outcomes, including depression. Consistent with this view, the level of loneliness has been reported to predict changes in depressive symptomatology (Cacioppo et al., 2010), and longitudinal evidence confirms that, in elderly adults, baseline loneliness scores are prospectively associated with greater severity of depressive symptoms at 12-year follow-up (Lee et al., 2021). At shorter timescales, higher levels of loneliness are also predictive of more severe social anxiety, paranoia and depression over 6 months (Lim et al., 2016). Converging evidence also links loss aversion to depression. Loss aversion is positively correlated with disease severity in patients with a diagnosis of major depressive disorder (Huh et al., 2016), and the relationship between this evidence and decreased striatal activity (Chandrasekhar Pammi et al., 2015) fits with the reduced responsiveness of the reward system in depressed patients (Keren et al., 2018).

Although other clinical conditions may involve high levels of loneliness and loss aversion, potentially with common alterations of shared neuro-cognitive mechanisms, the current clinical evidence remains sparse and heterogeneous. For this initial proposal, we therefore chose to restrict the scope of our analysis to depression.

## 5 Delineating the boundaries: loss aversion and loneliness as distinct constructs

Before turning to the broader implications of this novel perspective, we clarify that, despite our hypothesis of shared underlying neural mechanism, loss aversion and loneliness are distinct phenomena. This distinction is highlighted by several key differences.

First, although both phenomena have been associated with psychopathological conditions (including depression; Erzen and Çikrikci, 2018; Sediyama et al., 2020) and are negatively associated with well-being (Binte Mohammad Adib and Sabharwal, 2024; Koan et al., 2021), they differ in their typical expression. Loss aversion is a decision-making tendency observed in most individuals, and does not inherently entail clinical distress or economic dissatisfaction. Rather than representing a general tendency, loneliness is a condition experienced by individuals who perceive their social relationships as unsatisfactory or even defective. Therefore, while both phenomena may lead to “missed opportunities” (foregone gains in the case of loss aversion, and reduced access to social connection in the case of loneliness), only the latter is inherently associated with an explicit desire for (social) change. Thus, while loss aversion could be largely explained by a negativity bias within decision-making, loneliness represents a far more complex phenomenon that cannot be reduced only to a manifestation of this bias. Indeed, loneliness is primarily characterized by explicit social dissatisfaction, coupled with a cognitive profile of social negativity bias (e.g., hyper-vigilance for social threats) that paradoxically promotes withdrawal and isolation, thereby hindering social reconnection, and supporting the consolidation of social dissatisfaction (Bellucci et al., 2025).

Second, although both concepts involve a preferential processing of negative information, this tendency can be easily quantified in the economic context of loss aversion (e.g., *λ* ≈ 2), whereas it is more difficult to operationalize in the social domain of loneliness. The objective difference between outcomes such as −5 and +4 is constant in monetary decision-making, allowing to estimate the extent to which an individual’s subjective weighting deviates from the objective “expected value” (Knutson et al., 2005). In contrast, in social contexts it is far more difficult to assign precise objective values of negativity or positivity to events, and/or to quantify how subjective evaluations deviate from such a benchmark in lonelier individuals. This challenge is further complicated by the fact that hyper-vigilance toward negative stimuli in loneliness is often implicit, such that the explicit judgments may fail to capture the underlying implicit evaluations.

Reflecting this difference in formal tractability, loss aversion is typically operationalized as a well-defined asymmetry in the subjective weighting of losses and gains (negative and positive outcomes, respectively). By contrast, the hyper-vigilance for social threats in loneliness is assessed through more heterogeneous methods, including comparisons across social negative and social positive stimuli (following the negativity bias framework, e.g., Bangee et al., 2014; Shin and Kim, 2019), or between social negative stimuli and neutral (e.g., Arioli et al., 2025b) or non-social negative stimuli (e.g., Cacioppo et al., 2015), as well as group comparisons assessing the processing of negative social stimuli in lonely vs. non-lonely individuals (e.g., Du et al., 2022).

Finally, adopting an ecological perspective entails investigating behavior in contexts that allow to capture the entire process - from stimulus processing and evaluation to decision itself and its subsequent outcome - typically involving actual consequences. In the case of loss aversion, this requirement is relatively straightforward to meet, as most studies provide performance-based monetary incentives. Conversely, in the domain of loneliness, simulating a social context with genuine interpersonal exchanges and real consequences is extremely challenging, resulting both in experimental constraints and ethical concerns.

These distinctions do not limit the present perspective, and rather represent critical considerations for experimental design and result interpretations in future research.

## 6 Discussion and conclusion

Loss aversion and loneliness are well-established constructs in neuroscientific and psychological research. Traditionally, they have been studied as distinct phenomena grounded in separate domains, i.e., decision-making and social interaction, respectively. In this perspective paper, we challenge this separation and propose a novel unifying framework in which these constructs are partially supported by shared neuro-cognitive mechanisms.

We first reviewed loss aversion and loneliness separately, emphasizing the negativity bias as a common driver of both. We then detailed convergent cognitive and neural mechanisms, showing that both are associated with heightened attentional sensitivity to negative stimuli and/or experiences (be they financial losses or social threats), anticipation of adverse outcomes, and difficulties in emotion regulation. These cognitive and affective biases are reflected in specific neural signatures, involving structures associated with the processing of negative emotions, prospective rewards and punishments, as well as emotion regulation via cognitive control. From a clinical standpoint, high levels of loneliness and loss aversion also share convergent associations with depression, suggesting an overlapping profile at their extreme levels.

Following this overview, we discussed the key differences between the two constructs, particularly regarding how they are operationalized in experimental psychology and cognitive neuroscience. While loss aversion is a general tendency in decision-making that can be quantified using mixed-gamble paradigms, loneliness is a subjective experience of social disconnection, which makes valid—and yet ecological—assessment more complex and methodologically heterogeneous. Crucially, these distinctions are essential for evaluating the proposed analogy between the two constructs: rather than discouraging the search for their shared mechanisms, they help define conceptual boundaries and refine experimental approaches. In turn, this analysis enables a clearer conceptualization of both the common and distinctive facets across these aspects of human behavior likely shaped by the negativity bias. Accordingly, we should move beyond a purely “negative” lens focused on methodological obstacles, and rather emphasize the substantial gains of investigating their common basis, and the extent to which they outweigh the methodological challenges inherent in such interdisciplinary research.

Importantly, the present paper offers a theoretical perspective aimed at stimulating empirical research into the potential link between loss aversion and loneliness. Future studies in this field may employ both cross-sectional and longitudinal experimental paradigms to assess the common and distinctive factors shaping these phenomena, and their susceptibility to experiential, personological and/or demographic factors, as well as to experimental manipulations. A comprehensive empirical investigation of the shared mechanisms underlying the constructs of loss aversion and loneliness could not only provide novel insights into the clinical manifestations of depression, but also deepen our understanding of human behavior and of the neuro-cognitive mechanisms shaped by negativity bias and self-preservation across decision-making and social interaction.

## Data Availability

The original contributions presented in the study are included in the article/supplementary material, further inquiries can be directed to the corresponding author.
